# Cerebral and somatic venous oximetry in adults and infants

**DOI:** 10.4103/2152-7806.73316

**Published:** 2010-11-27

**Authors:** Erin A. Booth, Chris Dukatz, James Ausman, Michael Wider

**Affiliations:** 1 Medical Science, Somanetics Corporation, Troy, USA; 2 Department of Neurosurgery, David Geffen School of Medicine at UCLA, Los Angeles, CA, USA; 3 Department of Physiology, Wayne State University School of Medicine, Detroit, MI, USA

**Keywords:** INVOS, near infrared spectroscopy, noninvasive monitoring, Hemodynamic management, CO_2_ reactivity, tissue oxygenation

## Abstract

**Background:**

The development in the last decade of noninvasive, near infrared spectroscopy (NIRS) analysis of tissue hemoglobin saturation *in vivo* has provided a new and dramatic tool for the management of hemodynamics, allowing early detection and correction of imbalances in oxygen delivery to the brain and vital organs.

**Description:**

The theory and validation of NIRS and its clinical use are reviewed. Studies are cited documenting tissue penetration and response to various physiologic and pharmacologic mechanisms resulting in changes in oxygen delivery and blood flow to the organs and brain as reflected in the regional hemoglobin oxygen saturation (rSO_2_). The accuracy of rSO_2_ readings and the clinical use of NIRS in cardiac surgery and intensive care in adults, children and infants are discussed.

**Conclusions:**

Clinical studies have demonstrated that NIRS can improve outcome and enhance patient management, avoiding postoperative morbidities and potentially preventing catastrophic outcomes.

## NEAR INFRARED SPECTROSCOPY

Noninvasive, transcutaneous oximetry based on near infrared (NIR), diffuse reflectance spectroscopy has rapidly become a standard of care. While pulse oximetry is used to indicate arterial hemoglobin saturation, venous oximetry is used to measure tissue hemoglobin saturation in the capillary beds, reflecting O_2_ delivery and demand in the tissues. This simple, noninvasive technology is based on complex and in-depth chemical principles that have been refined over the last 80 years to provide unique and invaluable insights into oxygen biology.

The use of NIR light of 700–1000 nm wavelength for spectroscopic analysis of hemoglobin saturation *in vivo* is based on the fact that very few substances in tissue absorb NIR light, allowing for deeper light penetration. The absorption of electromagnetic radiation from X-ray to infrared wavelengths by chemical compounds is specific to the structure of the molecule being analyzed, with specific molecular bonds absorbing specific wavelengths of radiation. Accurate analysis of a compound normally requires that it be in a highly pure state without any contaminants, unless the contaminants do not absorb the specific wavelengths of radiation.

Metalloproteins with prophyrin rings are the only biologic structures that absorb much NIR light and the hemoglobin molar absorptivity at these overtone (harmonic) wavelengths is extremely low. The cytochrome enzymes absorb NIR light but the tissue concentration is an order of magnitude lower than hemoglobin. Additionally, myoglobin desaturation is limited, allowing the analysis of saturation to be hemoglobin specific.[49]

## NEAR INFRARED SPECTROSCOPY AND TISSUE OXYGENATION

Millikan coined the term “oximeter” in the 1940s[36] and it has been used ever since to indicate spectroscopic analysis of the ratio of oxy- and deoxyhemoglobin in tissue. Earlier, oximeters used visible light and red and blue filters to analyze the ratio of oxy- to deoxyhemoglobin, relying on the difference in color of these two compounds sometimes referred to as “chromophores”.

The early machines measured hemoglobin saturation in the blood just below the skin surface since visible light cannot penetrate tissue very far, but were surprisingly accurate for their simplicity. Though they were accurate, they could only provide information about capillary blood in the skin. It was not until the 1950s when Chance[8] advanced the concept of differential spectroscopy with NIR light *in vivo* and Jobsis in the 1970s[28] promoted its use for the estimation of hemoglobin saturation in tissue, which led to its practical clinical use becoming possible.

**Figure 1:**
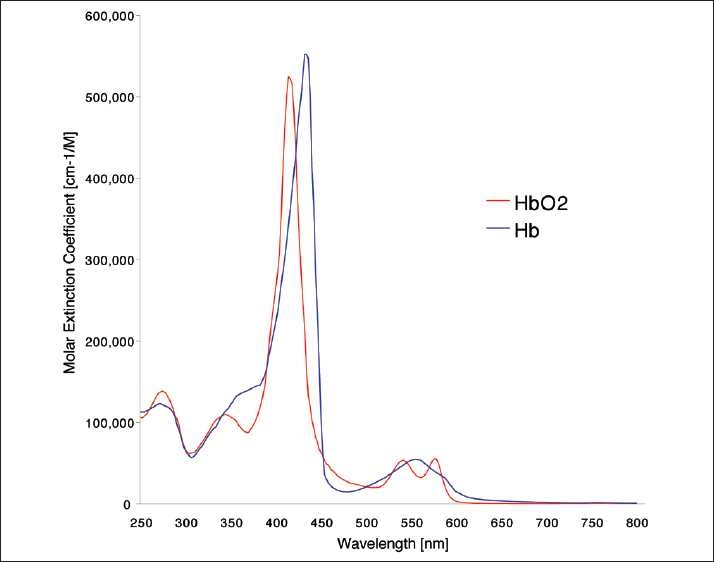
Oxy- and deoxyhemoglobin absorption spectra *in vitro*, showing strong absorption peaks at several ultraviolet and visible wavelengths but little absorption at overtone (harmonic) wavelengths above 650 nm (5) (reproduced with permission)

**Figure 2:**
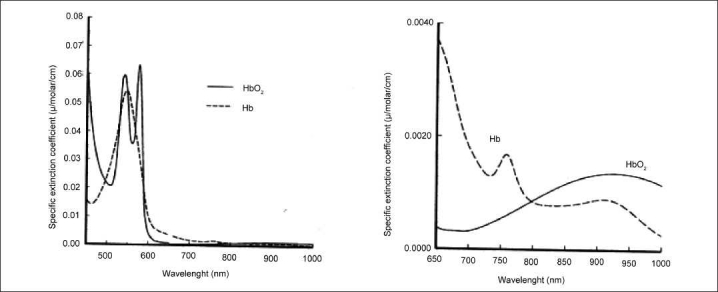
NIR absorption curve for oxy- and deoxyhemoglobin, showing the isobestic point at 805 nm and the dominance of deoxyhemoglobin absorption at wavelengths below 805 nm. The ratio of the absorption at 810 and 730 nm gives a measure of % oxygen saturation

The spectra in Figure 1 show that while hemoglobin absorption at shorter wavelengths is strong, there is very little absorption by oxy- and deoxyhemoglobin in the NIR region.[43] Hemoglobin as well as myoglobin and the cytochrome enzymes absorb light at NIR wavelengths but expansion of the Y axis is necessary to see the absorption spectra. Once this is done, a reproducible and specific absorption curve is obtained as shown in Figure 2.

The extremely low level of absorption of NIR light by hemoglobin is the dominant factor in achieving accurate measurements, requiring low light intensity in order to avoid overwhelming the signal and a very low level of noise in the system to produce a high signal to noise ratio. While benchtop co-oximeters utilize multiple wavelengths to differentiate various dyshemoglobins *in vitro*, noise reduction remains the most important factor in improving accuracy and precision *in vivo*.

## CLINICAL USE OF NEAR INFRARED SPECTROSCOPY

Near infrared spectroscopy (NIRS) has been used extensively over the past decade to monitor oxygen delivery to the brain and spine in adults, children and infants during cardiac and vascular surgeries. It has been shown to improve outcomes and prevent potentially catastrophic results from incidents such as accidental cannula misplacement.[7,12,14,19,38,51] Over the last several years, the use of NIRS to monitor both cerebral and somatic tissues in infants and children during cardiac surgery and in the intensive care unit (ICU) has grown steadily, contributing significantly to the management of hemodynamics.[2,5,16,22,31,42]

The accuracy of NIRS for monitoring brain O_2_ delivery has been validated by comparison to internal jugular vein hemoglobin saturation (SijvO_2_) levels reported in independent studies in adults, children and infants,[1,30,40] as well as in data submitted to the Food and Drug Administration (FDA) in support of product clearances.

There are a number of factors that need to be taken into account when validating the accuracy of any cerebral oximeter, including the significant incidence of gross anatomical variability of the vascular anatomy of the brain,[4] requiring placement of the sensor on the same side of the head as the jugular vein that was sampled. Compensation for skull and muscle is also essential and was developed from empirical observations of injection of indocyanine green dye in the internal and external carotid arteries,[26] ensuring that cerebral regional oxygen concentration (rSO_2_) is specific for brain.

**Figure 3:**
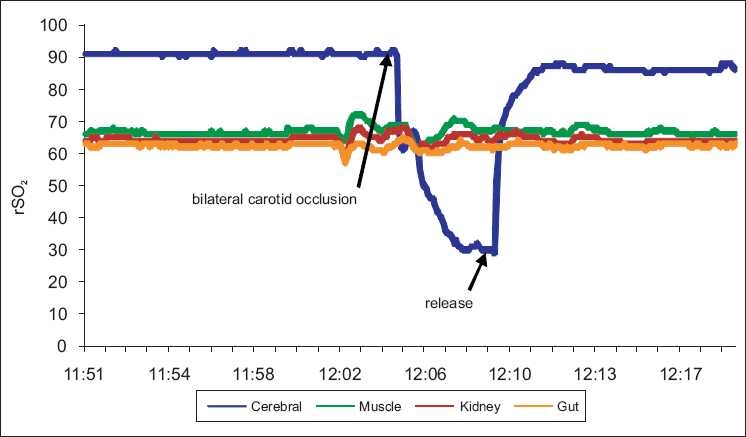
Rapid and dramatic C-rSO_2_ response to bilateral occlusion of the internal carotid arteries in a 4.2 kg piglet anesthetized with isoflurane (INVOS 5100C). Blood pressure remained unchanged and SpO_2_ was 100% for the entire experiment

Furthermore, rather than relying on global hypoxia, which affects all tissues, validation of specificity requires alteration of O_2_ delivery to that specific tissue. This was done for brain by altering partial pressure of carbon dioxide in the arterial blood (PaCO_2_) levels to increase cerebral perfusion in normal adults[30] and by internal carotid artery occlusion in an animal model[50] as shown in Figure 3. All physiologic parameters, including oxygen saturation of arterial blood (SpO_2_), remained constant during these experiments, demonstrating the ability of NIRS to obviate occult changes in blood flow to the brain when all other clinical measures remain stable.

The issues of accuracy and precision are essential components of analytical techniques and are requisites for validating any monitoring technology but are different from target physiologic values. While pulse oximetry measures the saturation of arterial blood (SpO_2_) by monitoring the pulse interval and has a defined target value, tissue oximetry is a venous weighted measure and hence reflects the arteriovenous (AV) difference and the adequacy of oxygen delivery. Tissue oximetry provides a range of “normal” saturation that is associated with venous outflow which is patient specific and is known to have wide variability.[9]

**Figure 4:**
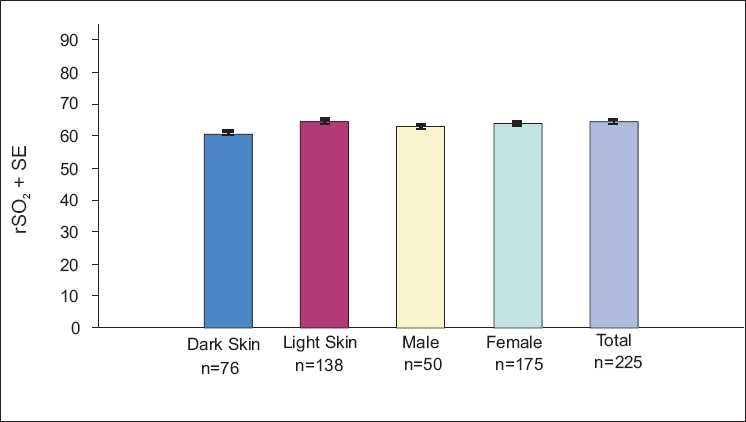
Cerebral rSO_2_ in normal, ambulatory adults (*n* = 226), showing the lack of impact of skin color and gender (INVOS 5100C) (IRB approved clinical study, 2008)

Studies reporting SijvO_2_ means and range in normal subjects and cardiac patients using co-oximetry of blood samples from the internal jugular vein[9,10,20,37] are consistent with cerebral rSO_2_ values reported in the literature [Table 1] and those obtained in a screen of normal ambulatory adults [Figure 4].

**Table 1:** Oxygen saturation values in the jugular bulb (SjO_2_) Chiergato *et al* (9)

	SjO_2_		
	Mean (95% CI)	Upper limit (95% CI)	Lower limit (95% CI)
Chiergato (2003)	57.1 (52.3–61.6)	69.5 (61.2–77.7)	44.7 (36.5–53.0)
Gibbs (1942)	62.0 (61.0–63.1)	69.4 (67.6–71.2)	54.6 (52.8–56.5)
Datsur (1963)	64.3 (62.4–66.2)	73.7 (70.4–76.9)	55.0 (51.7–58.2)

The range of SijvO_2_ determined by co-oximetry of blood samples from adult volunteers reported in three major studies. as referenced by Chiergato *et al* (9). Values are venous saturation and when converted to the field saturation (25:75 arterio:venous contribution) used in rSO_2_ (assuming 98% arterial saturation), the range in the “present series” study from 2003 (24) and in the report by Grubhofer *et al*. in 1998 (25) is identical to that seen with INVOS

It is critical to understand the value and accuracy of rSO_2_ to remember that it is venous weighted and hence responsive to all the physiologic factors that influence oxygen availability such as anatomical variability, hemoglobin dissociation, cardiac output, dyshemoglobinemias, blood pH, vascular permeability and metabolic demand, resulting in an AV difference. The greatest value of rSO_2_ monitoring results from the fact that it is impacted by all these variables and, hence, unlike SpO_2_, reflects the amount of O_2_ that was actually available to, and consumed by, the tissues.

**Figure 5:**
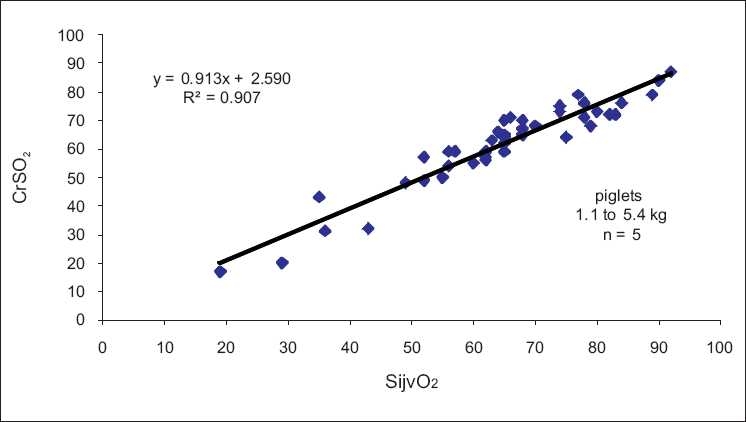
Regression analysis of cumulative rSO_2_ versus SijvO_2_ values during hypocapnic challenge or carotid occlusion (*n* = 5) in normal term piglets anesthetized with isoflurane (*r*^2^ = 0.9076)

Independent studies in humans and validation data for FDA clearance as well as data from animal studies shown in Figure 5 have demonstrated a high level of accuracy and specificity of cerebral rSO_2._

Somatic monitoring presents a greater challenge since the NIR light penetrates potentially thick muscle and fascial layers when applied to the body surface, making shallow compensation essential to limit the impact of intervening tissues. Animal experiments have demonstrated that when the skin to organ distance is less than 1.4 cm with the INVOS system (INVOS 5100C, Somanetics Corporation, Troy, MI, USA), changes in flow to the organ are reflected in immediate and sensitive change in the rSO_2_.

**Figure 6:**
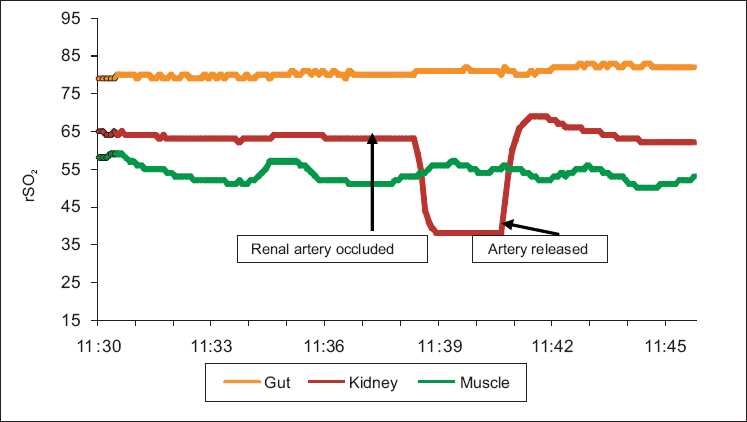
Rapid and dramatic rSO_2_ response to occlusion of the renal artery in a piglet anesthetized with isoflurane (INVOS 5100C). Blood pressure remained unchanged and SpO_2_ was 100% for the entire experiment

**Figure 7:**
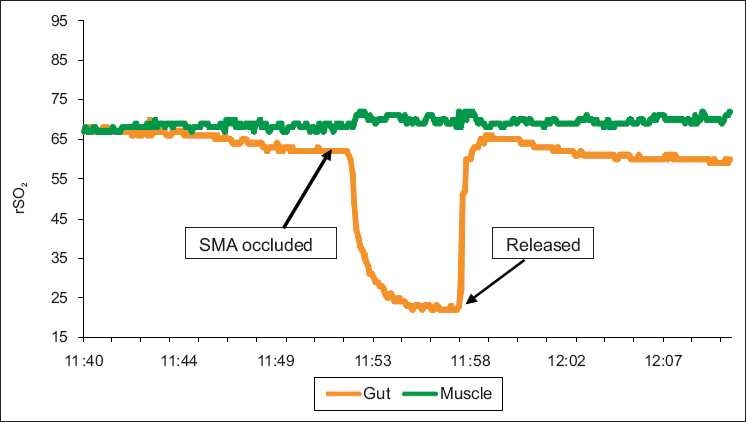
Rapid and dramatic rSO_2_ response to occlusion of the Superior mesenteric artery in a piglet anesthetized with isoflurane (INVOS 5100C). Blood pressure remained unchanged and SpO_2_ was 100% for the entire experiment

The rSO_2_ response to occlusion of the renal artery, stopping flow to the kidney in isoflurane anesthetized piglet, is shown in Figure 6 and the rSO_2_ response of the gut to occlusion of the superior mesenteric artery (SMA) is shown in Figure 7. The responses can be seen to be tissue specific with the other monitored tissues not changing in response to occlusion.

The specificity of the response to organ ischemia enables somatic rSO_2_ to be used as a measure of peripheral perfusion and oxygen delivery and has been demonstrated to be very sensitive to changes in organ blood flow.[25,50] The use of perirenal monitoring as an indicator of peripheral perfusion has been well established in hemodynamic management of infant and neonatal congenital heart patients in the operating room (OR) and ICU.[2,3,5,17,18,21,22,24,31,35,46]

Tissue oximetry is complex and highly informative but is not a standalone diagnostic. It is a valuable part of a differential diagnosis and has been shown to provide an early alert to occult hemodynamic changes not indicated by other physiologic parameters including arterial blood gases and mean arterial pressure.[21] The pioneering work of George Hoffman and the group at the Children’s Hospital of Wisconsin in the use of NIRS in congenital heart patients down to 1 kg has been successful in improving hemodynamic management, resulting in improved outcomes[29] in these patients.

The relationship between cerebral and somatic rSO_2_ has been defined and reduced to practice in congenital heart surgery and the cardiovascular intensive care unit (CVICU) with the perirenal rSO_2_ kept higher than cerebral to ensure adequate peripheral perfusion[47] where possible. Renal values for normal term infants are higher than cerebral values,[6] while preterm neonates have been reported to have renal values closer to cerebral rSO_2_. Preterm gut rSO_2_ is lower and more variable,[34] consistent with ultrasound studies of SMA flow in premature neonates.[41]

Cerebral saturation thresholds for intervention have been established in adults and neonates based on clinical[3,13,17,18,23,33,35,45,48] and animal[27,32] data indicating that significant neural damage can occur when cerebral rSO_2_ is below 40% for more than 180 or 30 minutes, respectively. Though prolonged values below 40% should always be of concern, impact on outcome can be patient specific due to the potential for ischemic preconditioning.[44] As can be seen in Table 1, a small percentage of normal adults have low jugular vein saturations without apparent physiologic deficits and 1.3% of the normal, ambulatory subjects included in Figure 4 had low C-rSO_2_. Setting preoperative baselines has become an essential part of NIRS to ensure that the small percentage of patients with tolerated low saturations is managed properly.

The management of hemodynamics in neonates presents a significant challenge. Blood pressure is adjusted with pressors and inotropes to the gestational age, which is standard of practice in many NICUs but is not well substantiated in relation to outcome.[11,15] Tissue- and drug-specific rSO_2_ response to pressors and inotropes has been reported,[39] demonstrating that simple targeting of pressure is not effective in guaranteeing adequate perfusion balancing between the brain and somatic organs.

The animal experiments cited above demonstrated drug specific responses in oxygen delivery between the brain and the kidney, gut and muscle, which would not be obvious from other clinical measures since the pressure and SpO_2_ remained unchanged. Simple adjustment of the mean arterial pressure to improve circulation has the potential to expose patients to the risk of increased cerebral flow, potentially leading to intracranial bleeds or conversely to hypoxic conditions from vasoconstriction that could cause vascular or neuronal damage.

NIRS holds great promise for managing the impact of pressors and inotropes in infants and neonates to ensure that peripheral vasoconstriction or inotropic stimulation results in appropriate perfusion balancing and does not cause organ morbidities while resulting in too much or too little blood flow to the brain. Conversely, there will be situations where it is necessary to deny the visceral organs to provide enough O_2_ to prevent neurological compromise despite the cost of organ damage. NIRS currently provides the only means of monitoring the distribution of perfusion in these instances.

While cerebral rSO_2_ below 40% has been shown to present significant risk as discussed above, there is no target level rSO_2_ for the organs. Multisite monitoring, however, provides insight into the tissue response to interventions allowing the clinician to asses the impact of those interventions on the distribution of blood flow.

**Figure 8:**
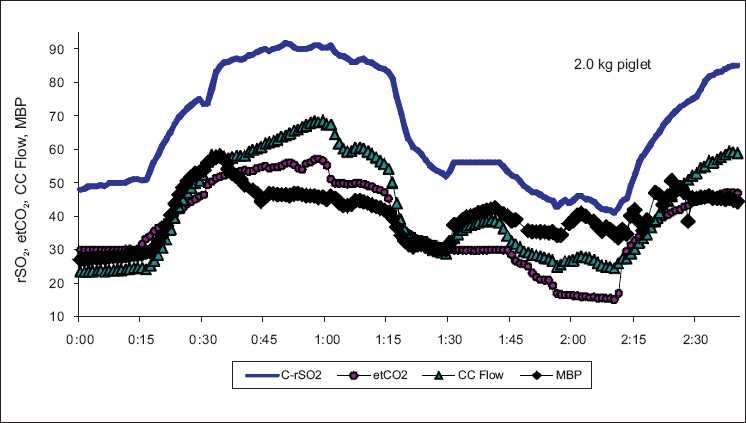
C-rSO_2_ tracking of cerebral O_2_ delivery response to hypercapnia followed by hypocapnia in a 2.0 kg, term, normothermic piglet, demonstrating the ability of NIRS to track the cerebral blood flow response to changes in CO_2_ (CC flow, common carotid flow; MBP, mean blood pressure; etCO_2_, end tidal CO_2_). SpO_2_ of 100% and blood pressure remained constant through the entire experiment. Periodic blood gas analysis was run to validate the etCO_2_

In addition to hemodynamic management, NIRS can provide insight into potential cerebral perfusion response to changes in ventilation. Premature neonate’s lungs are frequently kept functional with high frequency ventilation or continuous positive airway pressure, both of which can impact PaCO_2_ levels. Normal SpO_2_ does not ensure appropriate oxygen delivery to the brain which is responsive to changes in circulating CO_2_. C-rSO_2_ reflects blood flow to the brain, and hence O_2_ delivery, and responds rapidly to changes in PaCO_2_ as shown in Figure 8. Abrupt changes in C-rSO_2_ following ventilation changes can be used to direct the clinician to run a blood gas to protect against unanticipated CO_2_ change.

## CONCLUSIONS

Advances in the use of NIRS to determine cerebral and somatic oxygen delivery has provided a new and powerful tool for obviating frequently occult changes in oxygen biology that can result in serious morbidities and even death. The information provided by NIRS is a significant and unique addition to hemodynamic management and has been shown to improve the outcome and reduce the length of stay. While a threshold for cerebral rSO_2_ has been established in children and adults, the use of somatic monitoring provides a new component to differential diagnosis and intervention. The inclusion of multisite rSO_2_ in a diagnostic assessment provides insight into perfusion distribution and directs the clinician to assess the potential causes of change in cerebral or somatic O_2_ delivery, enhancing patient management and improving outcomes.

## References

[1] Abdul-Khaliq H, Troitzsch D, Berger F, Lange PE (2000). Comparison of regional transcranial oximetry with NIRS and jugular venous bulb oxygen saturation. Biomed Tech (Berl).

[2] Andropoulos DB, Stayer SA, Diaz LK, Ramamoorthy C (2004). Neurological monitoring for congenital heart surgery. Anesth Analg.

[3] Austin EH, Edmonds HL, Auden SM, Seremet V, Niznik G, Sehic A (1997). Benefit of neurophysiological monitoring for pediatric cardiac surgery. J Thorac Surg.

[4] Beards SC, Yule S, Kassner A, Jackson A (1998). Anatomical variation of cerebral venous drainage: The theoretical effect in jugular bulb blood samples. Anesthesia.

[5] Berens RJ, Stuth EA, Robertson FA, Jaquiss RD, Hoffman GM, Cava JR (2006). NIRS monitoring during pediatric aortic coarctation repair. Paediatr Anaesth.

[6] Bernal NP, Hoffman GM, Ghanayem NS, Arca MJ (2010). Cerebral and somatic NIRS in normal newborns. J Pediatr Surg.

[7] Casati A, Fanelli G, Pietropaoli P, Proietti R, Tufano R, Montanini S (2006). Monitoring cerebral oxygen saturation in elderly patients undergoing general abdominal surgery: A prospective cohort study. Eur J Anaesthesiol.

[8] Chance B (1951). Rapid and sensitive spectrophotometry III: A double beam apparatus. Rev Sci Instrum.

[9] Chieregato A, Calzolari F, Trasforini G, Targa L, Latronico N (2003). Normal jugular bulb oxygen saturation. J Neurol Neurosurg Psychiatry.

[10] Croughwell ND, White WD, Smith LR, Davis D, Glower DD, Reves JG (1995). Jugular bulb saturation and mixed venous saturation during cardiopulmonary bypass. J Card Surg.

[11] Dempsey EM, Barrington KJ (2007). Treating hypotension in the preterm neonate: When and with what: A critical and systematic review. J Perinatol.

[12] Denault A, Deschamps A, Murkin J (2007). A proposed algorithm for the intraoperative use of cerebral NIRS. Semin Cardiothorac Vasc Anesth.

[13] Dent CL, Spaeth JP, Jones BV, Schwartz SM, Glauser TA, Hallinan B (2006). Brain magnetic resonance imaging abnormalities after the Norwood procedure using regional cerebral perfusion. J Thorac Cardiovasc Surg.

[14] Edmonds HL, Ganzel BL, Austin EH (2004). Cerebral oximetry for cardiac and vascular surgery. Semin Cardiothorac Vasc Anesth.

[15] Fanaroff JM, Fanaroff AA (2006). Blood pressure disorders in the neonate: Hypotension and hypertension. Semin Fetal Neonatal Med.

[16] Farouk A, Karimi M, Ostrowsky J, Siwik E, Hennien H (2008). Cerebral regional oxygenation during aortic coarctation repair in pediatric population. Eur J Cardiothorac Surg.

[17] Fenton KN, Freeman K, Glogowski K, Fogg S, Duncan KF (2005). The significance of baseline cerebral oxygen saturation in children undergoing congenital heart surgery. Am J Surg.

[18] Ghanayem NS, Mitchell ME, Tweddell JS, Hoffman GM (2006). Monitoring the brain before, during and after cardiac surgery to improve long-term neurodevelopmental outcomes. Cardiol Young.

[19] Gottlieb EA, Fraser CD, Andropoulos DB, Diaz LK (2006). Bilateral monitoring of cerebral oxygen saturation results in recognition of aortic cannula malposition. Paediatr Anaesth.

[20] Grubhofer G, Lassnigg AM, Schneider B, Rajek MA, Pernerstorfer T, Heismayr MJ (1998). Jugular venous bulb oxygen saturation depends on lood pressure during CPB. Ann Thorac Surg.

[21] Hanson SJ, Berens RJ, Havens PL, Kim MK, Hoffman GM (2009). Effect of volume resuscitation on regional perfusion in dehydrated pediatric patients as measured by 2 site NIRS. Pediatr Emerg Care.

[22] Hoffman GM, Stuth EA, Jaquiss RD, Vanderwal PL, Staudt SR, Troshynski TJ (2004). Changes in cerebral and somatic oxygenation during stage 1 paliation of hypoplastic left heart syndrome using regional perfusion. J Cardiothorac Surg.

[23] Hoffman GM, Ghanayem NS, Mussatto KA, Musa N (2005). Perioperative perfusion assessed by somatic NIRS predicts postoperative renal dysfunction. Anesthesiology.

[24] Hoffman GM, Ghanayem NS, Tweddell (2005). Noninvasive assessment of cardiac output. Semin Thorac Cardiovasc Surg Pediatr Card Surg Annu.

[25] Hoffman GM, Wider MD (2008). Organ specificity of NIRS rSO_2_ measurements during regional ischemia in piglets. Anesthesiology.

[26] Hongo K, Kobayashi S, Okudera H, Hokama M, Nakagawa F (1995). Noninvasive cerebral optical spectroscopy; depth resolved measurements of cerebral hemodynamics using indocyanine green. Neurol Res.

[27] Hou X, Ding H, Teng Y, Shou C, Tang X, Li S et al (2007). Research on the relationship between brain anoxia at different regional oxygen saturations and brain damage using NIRS. Physiol Meas.

[28] Jobsis FF (1977). Noninvasive, infrared monitoring of cerebral and myocardial sufficiency and circulatory parameters. Science.

[29] Johnson BA, Hoffman GM, Tweddell JS, Cava JR, Basir M, Mitchell ME (2009). NIRS in neonates before palliation of HLHS. Ann Thorac Surg.

[30] Kim MB, Ward DS, Cartwright CR, Kolano J, Chelebowski S, Henson LC (2000). Estimation of jugular venous O2 saturation from cerebral oximetry or arterial O2 saturation during isocapnic hypoxia. J Clin Monit.

[31] Kirshbom PM, Forbess JM, Kogon BE, Simsic JM, Kim DW, Raviele AA (2007). Cerebral NIRS is a reliable marker of systemic perfusion in awake single ventricle children. Pediatr Cardiol.

[32] Kurth CD, Levy WJ, McCann J (2002). Near-infrared spectroscopy cerebral oxygen saturation thresholds for hypoxia-ischemia in piglets. J Cereb Blood Flow Metab.

[33] Lemmers PM, Toet MC, van Bel F (2008). Impact of PDA and subsequent therapy with indomethacin on cerebral oxygenation. Pediatrics.

[34] McNeill S, Gatenby JC, McElory S, Engelhardt B (2010). Normal cerebral, renal and abdominal regional saturations using NIRS in preterm infants. J Perinatol.

[35] McQuillen PS, Nishimoto MS, Bottrell CL, Fineman LD Hamrick SE, Glidden DV (2007). Regional and central venous oxygen saturation monitoring following pediatric cardiac surgery. Pediatr Crit Care Med.

[36] Millikan GA (1942). The oximeter, an instrument for measuring the oxygen saturation of the arterial blood in man. Rev Sci Instrum.

[37] Miyoshi S, Morita T, Kadoi Y, Goto F (2005). Analysis of the factors related to a decrease in jugular venous oxygen saturation in patients with diabetes mellitus during bypass. Surg Today.

[38] Murkin JM, Adams SJ, Novick RJ, Quantz M, Bainbridge D, Iglesias I (2007). Monitoring brain oxygen saturation during coronary artery bypass surgery: A randomized, Prospective Study. Anesth Analg.

[39] Nachar-Hidalgo RA, Booth EA, Drake SL, Wider MD, Seri I Effects of dose-escalation studies with dopamine, dobutamine, epinephrine and milrinone on regional tissue oxygen saturation in normotensive, euvolemic neonatal piglets.

[40] Nagdyman N, Ewert P, Peters B, Miera O, Fleck T, Berger F (2008). Comparison of different NIRS cerebral oxygenation indices with central venous and jugular oxygenation saturation in children. Paediatr Anaesth.

[41] Papacci P, Giannantonio C, Cota F, Latella C, Semeraro CM, Fioretti M (2009). Neonatal color Doppler US study: Normal values of abdominal blood flow velocity. Pediatr Radiol.

[42] Phelps HM, Mahle WT, Kim D, Simsic JM, Kirshbom PM, Kanter KR (2009). Postop cerebral oxygenation in HLHS after the Norwood procedure. Ann Thorac Surg.

[43] Poloschek CM, Sutter EE (2002). The fine structure of multifocal ERG topographies. J Vis.

[44] Shpargel KB, Jalabi W, Jin Y, Dadabayev A, Penn MS, Trapp BD (2008). Preconditioning paradigms and pathways in the brain. Cleve Clin J Med.

[45] Singer I, Edmonds HL (2000). Tissue oximetry for the diagnosis of neurally mediated syncope. Pacing Clin Electrophysiol.

[46] Tina LG, Frigiola A, Abella R, Artale B, Puleo G, Angelo SD (2009). NIRS in healthy preterm and term newborns: Correlation with GA and standard monitoring. Curr Neurovasc Res.

[47] Tweddell JS, Ghanayem N, Hoffman G (2010). NIRS is Standrad of Care for postoperative management. Semin Thorac Cardiovasc Surg Pediatr Card Surg Annu.

[48] Villafane J, Edmonds HL (2001). Volume expansion prevents tilt-table induced syncope. Cardiol Young.

[49] Ward KR, Ivatury RR, Barbee RW, Terner J, Pittman R, Filho IP (2006). NIRS for Evaluation of the Trauma Patient: A technology review. Resuscitation.

[50] Wider MD (2009). Hemodynamic management and regional hemoglobin oxygen saturation of the brain, kidney and gut. J Perinatol Neonatol.

[51] Yao F, Tseng C, Ho C, Levin S, Illner P (2004). Cerebral oxygen desaturation is associated with early postoperative neuropsychological dysfunction in patients undergoing cardiac surgery. J Cardiothorac Vasc Anesth.

